# Supplementary material for: The Systems Biology Research Tool: evolvable open-source software
Source: BMC Syst Biol. 2008 Jun 29;2:55. doi: 10.1186/1752-0509-2-55 (PMC2446383; doi:10.1186/1752-0509-2-55)
Supplement: Additional file 1 — SBRT Archive. An archive of the current version of the Systems Biology Research Tool. [file 1752-0509-2-55-S1.zip › sbrt-1.4.0/doc/developers_guide/api/sbrt/kernel/fba/comp/SimpleFluxIntervalComputer.html]

SimpleFluxIntervalComputer


|  |  |  |
| --- | --- | --- |
| **Overview** \| **Package** \| **Class** \| **Use** \| **Tree** \| **Deprecated** \| **Index** \| **Help** | |  |
| **PREV CLASS**   **NEXT CLASS** | **FRAMES**    **NO FRAMES**     **All Classes** |  |
| SUMMARY: NESTED \| FIELD \| CONSTR \| METHOD | DETAIL: FIELD \| CONSTR \| METHOD |  |


---


## sbrt.kernel.fba.comp Class SimpleFluxIntervalComputer

```
java.lang.Object
  sbrt.kernel.fba.comp.AbstractCvComputer
      sbrt.kernel.fba.comp.AbstractFluxIntervalComputer
          sbrt.kernel.fba.comp.SimpleFluxIntervalComputer
```

**All Implemented Interfaces:**: FluxCvComputer, FluxIntervalComputer

---

``` public class SimpleFluxIntervalComputer extends AbstractFluxIntervalComputer ```

This class is used to compute flux intervals in the simplest possible way. An
interval is computed by minimizing and maximizing the flux of a given
reaction. If a flux is unbounded, as they sometimes are,
Double.NEGATIVE\_INFINITY and
Double.POSITIVE\_INFINITY are used to denote the flux bounds.

**Author:**
:   This class was written and documented by Jeremiah Wright while in the
    Wagner lab.

---

**Field Summary**

**Fields inherited from class sbrt.kernel.fba.comp.AbstractFluxIntervalComputer**

`ZERO_CUTOFF`


**Constructor Summary**

`SimpleFluxIntervalComputer(FbaOptComputer computer,
double zeroCutoff)`
  
          Constructs a new flux interval computer.


| **Method Summary** | |
| --- | --- |
| `java.util.Map<java.lang.String,Interval>` | `getIntervals()`             Computes and returns the intervals of every flux in the fluxome used to construct this flux interval computer. |
| `java.util.Map<java.lang.String,Interval>` | `getIntervals(java.util.Collection<java.lang.String> rxnNames)`             Computes and returns the flux intervals of the reactions with the provided names. |
| `java.util.Map<java.lang.String,java.lang.Double>` | `getMaximumFluxes(java.util.Collection<java.lang.String> rxnNames, MutableProgressMonitor progress)`             Computes and returns the maximum fluxes of the reactions with the provided names. |
| `ProgressMonitor` | `getProgress()`             Returns a progress monitor for this flux interval computer. |

**Methods inherited from class sbrt.kernel.fba.comp.AbstractFluxIntervalComputer**

`getBoundedMax, getBoundedMax, getBoundedMaxWithReverseConstrained, getBoundedMin, getBoundedMin, getConstraintTolerance, getFluxConstraint, getFluxConstraints, getInterval, getIntervals, getMaximum, getMaximum, getMaxWithReverseConstrained, getMinimum, getMinimum, getReverseRxnNames, getRxnExprConstraint, getRxnExprConstraints, getZeroCutoff, restoreFluxConstraint, restoreFluxConstraints, restoreFluxConstraints, restoreRxnExprConstraint, restoreRxnExprConstraints, restoreRxnExprConstraints, setFluxConstraint, setFluxConstraints, setRxnExprConstraint, setRxnExprConstraints`

**Methods inherited from class sbrt.kernel.fba.comp.AbstractCvComputer**

`checkFluxConstraint, checkNumber, checkRxnExpr, checkRxnExprConstraint, checkRxnName, checkRxnNames, getFluxome, getRxnExpr, getRxnExpr`

**Methods inherited from class java.lang.Object**

`clone, equals, finalize, getClass, hashCode, notify, notifyAll, toString, wait, wait, wait`

**Methods inherited from interface sbrt.kernel.fba.comp.FluxCvComputer**

`getFluxome`

**Constructor Detail**

### SimpleFluxIntervalComputer

```
public SimpleFluxIntervalComputer(FbaOptComputer computer,
                                  double zeroCutoff)
```

:   Constructs a new flux interval computer.

    **Parameters:**: `computer` - the computer with which all optimizations will be computed.: `zeroCutoff` - the amount by which a computed lower bound can *exceed* a computed upper bound.


**Method Detail**

### getProgress

```
public ProgressMonitor getProgress()
```

:   Returns a progress monitor for this flux interval computer.

    :   **Returns:**: a progress monitor for this flux interval computer.

---


### getIntervals

```
public java.util.Map<java.lang.String,Interval> getIntervals()
```

:   Computes and returns the intervals of every flux in the fluxome used to
    construct this flux interval computer.

    :   **Returns:**: reaction names mapped to the computed interval of their corresponding flux.

---


### getIntervals

```
public java.util.Map<java.lang.String,Interval> getIntervals(java.util.Collection<java.lang.String> rxnNames)
```

:   Computes and returns the flux intervals of the reactions with the
    provided names.

    :   **Parameters:**: `rxnNames` - a collection of reaction names. **Returns:**: reaction names mapped to the computed interval of their corresponding flux.

---


### getMaximumFluxes

```
public java.util.Map<java.lang.String,java.lang.Double> getMaximumFluxes(java.util.Collection<java.lang.String> rxnNames,
                                                                         MutableProgressMonitor progress)
```

:   Computes and returns the maximum fluxes of the reactions with the
    provided names. The provided progress monitor is incremented after
    each maximum flux is computed.

    :   **Parameters:**: `rxnNames` - a collection of reaction names.: `progress` - the progress monitor to be incremented. **Returns:**: reaction names mapped to their corresponding maximum flux.


---


|  |  |  |
| --- | --- | --- |
| **Overview** \| **Package** \| **Class** \| **Use** \| **Tree** \| **Deprecated** \| **Index** \| **Help** | |  |
| **PREV CLASS**   **NEXT CLASS** | **FRAMES**    **NO FRAMES**     **All Classes** |  |
| SUMMARY: NESTED \| FIELD \| CONSTR \| METHOD | DETAIL: FIELD \| CONSTR \| METHOD |  |


---
